# Pregnancy and the risk of NICU admissions in Nandom Municipality of Ghana: A cross‐sectional retrospective study

**DOI:** 10.1002/hsr2.1070

**Published:** 2023-01-18

**Authors:** Maroun Soribang Ziem, Fidelis Adam Saaka, Ezekiel Kofi Vicar, Eugene Dogkotenge Kuugbee, Akosua Bonsu Karikari, Sebastian Yidana Ninimiya, Juventus Benogle Ziem, Williams Walana

**Affiliations:** ^1^ Department of Community Medicine, School of Medicine University for Development Studies Tamale Ghana; ^2^ Department of Microbiology, School of Medicine University for Development Studies Tamale Ghana; ^3^ Department of Molecular Medicine CKT‐UTAS Navrongo Ghana; ^4^ Department of Obstetric and Gynecology St. Theresa's Hospital Nandom Ghana; ^5^ Department of Clinical Microbiology and Immunology, School of Medicine and Dentistry CKT‐UTAS Navrongo Ghana

**Keywords:** Ghana, Nandom, neonatal intensive care unit, neonates, NICU, pregnancy

## Abstract

**Background:**

Neonatal intensive care units (NICU) are specialized units that provide medical attention to neonates, and thus have become a vital aspect in the provision of critical care to infants who are faced with special challenges following birth.

**Aim:**

To determine antepartum and intrapartum factors that predispose to NICU admissions in the Nandom Municipal of the Upper West Region of Ghana.

**Method:**

This was a cross‐sectional retrospective study, spanning from January 1, 2021 to December 31, 2021. Records covering 1777 women who were delivered or had their babies referred to the St. Theresa's Hospital in the Nandom Municipality were involved in the study. Descriptive statistics and multinomial logistic regression analysis were used to compare variables, and statistical significance was determined where the p‐value was less than 0.05.

**Results:**

From the study, the rate of NICU admission was 10.4%. There was a significant association between mothers who attended less than four antenatal sessions (*p* = 0.004) and admission to NICU. Nulliparous mothers (*p* = 0.027) and mothers who presented with multiple pregnancy (*p* < 0.001) were more likely to have their babies sent to NICU. Both preterm delivery (*p* < 0.001) and post‐term delivery (*p* < 0.001) were prone to admission to NICU. Also, instrumental delivery (*p* < 0.001), cesarean section (*p* < 0.001), low birth weight (*p* < 0.001), and male infants (*p* = 0.003) had an increased risk of being admitted to NICU. Furthermore, severe (*p* < 0.001) and moderate (*p* < 0.001) birth asphyxia in the first minute following delivery were significantly associated with NICU admission whereas severely asphyxiated babies at 5 min (*p* < 0.001) were associated with NICU admission.

**Conclusion:**

The study revealed a relatively high NICU admission rate in the study area, and the predictors are multifaceted. Tailored intervention programs aimed at curbing these predictors will be required to reduce the rate of NICU admissions in the Nandom Municipality of Ghana.

## 1 INTRODUCTION

Globally, particular attention had been given to reducing childhood mortality in a bid to achieve the Millennium Development Goal (MDG) 4; reducing under‐five mortality by two‐thirds between 1990 and 2015, and now encapsulated in the Sustainable Development Goal (SDG) number 3 which seeks to ensure healthy lives and promote well‐being for all at all ages.^1^, ^2^ According to the United Nations, between 1990 and 2015, the global under‐five mortality rate declined by more than half, dropping from 90 to 43 deaths per 1000 live births.^3^, ^4^

The current infant mortality rate for Ghana in 2022 is 31.768 deaths per 1000 live births, which represents a 2.95% decline from the 2021 estimate of 32.735 deaths per 1000 live births.^5^ Studies indicate that deaths within the neonatal period are responsible for the majority of mortalities in children under the age 5 years with a reported rate of 35% in the USA and up to 99% in certain low‐income countries.^3^, ^4^, ^6^ A child born in sub‐Saharan Africa was 10 times more likely to die in the first month than a child born in a high‐income country, while a child born in South Asia was nine times more likely to die.^4^ Across countries, the risk of dying in the first month of life was about 56 times higher in the highest‐mortality country than in the lowest‐mortality country.^4^ The majority of these neonatal deaths are mostly due to conditions that could be prevented or treated when there is access to simple and affordable interventions.^7^

Such interventions have been identified to include vaccination of women of childbearing age against tetanus, healthy and professional delivery care provision, new‐born resuscitation skills, ensuring proper exclusive breastfeeding, ensuring clean and infection‐free umbilical cord, and effective uptake of requisite new‐born vaccines.^8^ Others include pre‐pregnancy interventions, family planning, prevention and management of sexually transmitted infections including HIV, folic acid fortification, pregnancy interventions, and childbirth interventions.^9^

Several factors such as the patient's socio‐demographic factors and maternal and neonatal‐related factors are known to contribute directly or indirectly to neonatal morbidity and subsequent NICU admissions.^8^, ^9^ Many of these factors when predictable in a given healthcare setting, do contribute to the improvement of neonatal healthcare.^10^ Maternal factors such as parity, mode of delivery, prevailing medical conditions, and pregnancy‐related complications such as antepartum hemorrhage are associated with neonatal admission to the NICU.^11^ Similarly, neonatal factors shown to be associated with admission to the NICU include prematurity, low birth weight, intrauterine growth retardation (IUGR), birth asphyxia, meconium aspiration syndrome, and congenital anomalies.^10^

Facilities to set up a well‐functioning NICU are scarce especially in developing countries. Where they are available, factors associated with neonatal admission to the NICU appear to be variable and could be dependent on the healthcare environment. Knowledge of these associated factors will provide useful information regarding resourcing, preparedness and smooth operation of the NICUs. This study, therefore, explored the “feto‐maternal” predictors of NICU admissions in the Nandom Municipality of the Upper‐West region of Ghana.

## 2 METHODOLOGY

### 2.1 Study area

The Nandom Municipality is one of the 11 administrative municipalities and districts that make up the Upper West Region of Ghana. The municipality constitutes the north‐western corner of the Upper West Region of Ghana, geographically defined by longitudes 2°25 W and 2°45 W and latitudes 10°20 N and 11°00 S. The north‐western boundary of the municipality is also the international boundary between Ghana and the republic of Burkina Faso, whereas to the East, it shares boundary with the Lambussie district and to the south the Lawra Municipality. The municipality has a total land mass of 404.6 square km constituting approximately, 3.1% of the Upper West Region's total land mass. The Municipality is rural as 86% of the members of the municipality's 84 communities live in rural areas, and the population density is approximately 133 per square km.

### 2.2 Study design and data collection

A retrospective design was employed to carry out this study. The study population included all births delivered at St. Theresa's Hospital or referred to the hospital. The period reviewed covered January 1, 2021 to December 31, 2021. To extract relevant data from the maternal delivery record books, a structured questionnaire was designed based on the captioned information in the records book. The questionnaire was coded on a Google Form for uniformity and pretesting. After the necessary corrections to the tool, relevant socio‐demographic, maternal, and neonatal data for the study were extracted onto a Google Form. In all, a total of 1777 births were reviewed over the study period, and thus served as the sample size.

### 2.3 Data analysis

The data collected via the Google Form was extracted into Microsoft Excel for data cleaning, and subsequently exported into the IBM Statistical Package for Social Sciences (SPSS) software version 26 for analysis. The variables were categorized and descriptive statistics applied. Where applicable, multinomial logistic analysis was done for differences between the categorical variables and outcome variables, and a p‐value below 0.05 was considered statistically significant.

### 2.4 Ethical consideration

This study was approved by the University for Development Studies Institutional Review Board (UDS/RBI/107/22). Permission was granted by the hospital through the Medical Superintendent and the Maternity Ward In‐charge to carry out the study using the hospital's records. The data were anonymously collected and kept confidential.

## 3 RESULTS

From January 1 to December 31, 2021, 1777 babies were put to birth by 1772 mothers in St. Theresa's hospital, Nandom, of which, 10.4% (185/1777) of the babies were admitted to the NICU of the hospital. Mothers' ages ranged from 13 to 48 years, with a mean age of 28 ± 6 years, and 57.1% (11,014) of them were within the age range of 20–30 years. The literacy rate among 1385 mothers was 71.0% with a majority of 41.7% of the educated ones attaining Junior High School level of education. While 68.0% (924/1359) of the mothers were self‐employed, only 7.6% (103/1359) of them were engaged in formal employment. The mothers' demographic characteristics did not influence the babies' admission to the NICU (Table 1).

**Table 1:** Mothers' socio‐demographic characteristics and antenatal attendance

Variable	Total	Not admitted to NICU N (%) (*ref)*	Admitted to NICU N (%)	Exp (CI) (95%)	*p* Value
Age (years)					
<20	193 (10.9)	167 (86.5)	26(13.5)	1.305 (0.635–2.683)	0.468
20–30	1014 (57.2)	908 (89.5)	106 (10.5)	1.025 (0.595–1.766)	0.930
31–35	305 (17.2)	278 (91.1)	27 (8.9)	0.841 (0.439–1.612)	0.602
>35 (***ref***)	259 (14.6)	233 (90.0)	26 (10.0)		
Total	1772 (100)	1586 (89.5)	186 (10.5)		
Educational attainment					
No formal education	402 (29.0)	358 (89.1)	44 (10.9)	0.841 (0.349–2.024)	0.699
Primary	202 (14.6)	180 (89.1)	22 (10.9)	0.819 (0.322–2.079)	0.674
JHS	410 (29.6)	356 (86.8)	54 (12.9)	1.071 (0.460–2.491)	0.874
SHS	233 (16.8)	203 (87.1)	30 (12.9)	1.061 (0.471–2.390)	0.886
Tertiary (***ref**)*	138 (10.0)	121 (87.9)	17 (12.3)		
Total	1385 (100)	1218 (87.9)	167 (12.1)		
Job category					
Housewife	220 (16.2)	188 (85.5)	32 (14.5)	1.001 (0.516–1.944)	>0.99
Self‐employed	924 (68.0)	818 (88.5)	106 (11.5)	1.315 (0.734–2.358)	0.357
Student	112 (8.2)	99 (88.4)	13 (11.6)	1.298 (0.585–2.878)	0.521
Formal employment (***ref***)	103 (7.6)	88 (85.4)	15 14.6)		
Total	1359 (100)	1193 (87.8)	166 (12.2)		

Abbreviations: JHS, Junior High School;  (***ref***), reference category; SHS, Senior High School.

Although 92.8% (1225/1644) of mothers achieved the recommended 4 or more ANC attendance before delivery, ANC attendance significantly influenced the proportion of babies admitted to the NICU. A higher proportion of babies whose mothers did not attain the minimum ANC attendance (<4 times) was admitted to the NICU (19.3%; *p* = 0.004) compared to those whose mothers achieved the recommended ANC attendance. The odds of NICU admissions among babies whose mothers failed to adhere to the minimum standards of four times ANC attendance before delivery was twice compared to those who achieved the recommended 4 or more ANC attendance (Table 2).

**Table 2:** Mothers' gynecologic and obstetric history

Obstetric history		Total		Not admitted to NICU N (%) *ref*		Admitted to NICU N (%)		Exp (CI) (95%)		*p* Value
Antenatal attendance										
<4		119 (7.2)		96 (80.7)		23 (19.3)		2.166 (1.281–3.661)		0.004
≥4 (***ref***)		1525 (92.8)		1378 (90.4)		147 (9.6)				
Total		1644 (100)		1474 (89.7)		170 (10.3)				
Parity										
Nulliparous		588 (50.1)		506 (86.1)		82 (13.9)		3.666 (1.159–11.593)		0.027
Primiparous		383 (32.7)		353 (92.2)		30 (7.8)		0.959 (0.331–2.781)		0.939
Multiparous		653 (55.7)		596 (91.3)		57 (8.7)		0.955 (0.416–2.190)		0.913
Grand multiparous ***(ref)***		149 (12.7)		133 (89.3)		16 (10.7)				
Total		1773 (100)		1588 (89.6)		185 (10.4)				
Gravidity										
Primigravida		521 (44.5)		452 (86.8)		69 (13.2)		0.406 (0.148–1.108)		0.078
Multigravida		894 (76.3)		817 (91.4)		77 (8.6)		0.753 (0.368–1.540)		0.437
Grand multigravida ***(ref)***		356 (30.4)		318 (89.3)		38 (10.7)				
Total		1771 (100)		1587 (89.6)		184 (10.4)				
Type of pregnancy										
Single ***(ref)***		1666 (94.4)		1506 (90.4)		160 (10.6)				
Multiple		99 (5.6)		75 (75.8)		24 (32.0)		0.325 (0.167–0.633)		<0.001
Total		1765 (100)		1581 (89.6)		184 (11.6)				
Gestational age										
Preterm		276 (16.1)		210 (76.1)		66 (31.4)		3.981 (2.827–5.606)		<0.001
Term ***(ref)***		1394 (81.6)		1292 (92.7)		102 (7.9)				
Post‐term		39 (2.3)		27 (69.2)		12 (44.4)		5.630 (2.770–11.442)		<0.001
Total		1709 (100)		1529 (89.5)		180 (11.8)				
Labor onset										
Spontaneous		1447 (96.7)		1319 (91.2)		128 (9.7)		1.925 (0.592–6.258)		0.276
Induced ***(ref)***		50 (3.3)		46 (92.0)		4 (8.7)				
Total		1497 (100)		1365 (91.2)		132 (9.7)				
Mode of delivery										
Instrumental delivery		46 (2.6)		32 (69.6)		14 (43.8)		5.847 (2.960–11.548)		<0.001
Cesarean section		408 (23.1)		340 (83.3)		68 (20.0)		2.177 (1.545–3.068)		<0.001
Spontaneous vaginal delivery ***(ref)***		1312 (74.3)		1211 (92.3)		101 (8.3)				
Total		1766 (100)		1583		183				

Abbreviation: (***ref***), reference category.

By mode of delivery, 74.3% (1312) of the babies were delivered through spontaneous vaginal delivery (SVD), 2.6% (46) by instruments‐assisted delivery, whereas caesarian delivery was 23.1% (408). A significantly higher proportion of babies delivered through instruments assisted delivery (43.8% (14/46)) and through caesarian section (20.0% (68/408)) were admitted to the NICU, with odds of 6 and 2 respectively (Table 2).

Babies delivered at term gestation constituted 81.6% (1,394) while 16.1% (276) and 2.3% (39) of the babies were delivered preterm and post‐term respectively, yet in comparison to babies delivered at term, 44.4%^12^ of the post‐term babies had six times risk (*p* < 0.001) of being admitted to the NICU and 31.4% (66) of the preterm babies were admitted to the unit with four times risk (*p* < 0.001). Multiple babies per delivery were significantly associated with admission to the NICU compared to singletons. Although 94.4% (1666) of the babies were singletons, the proportion of multiple babies admitted to the unit was significantly higher with respect to the single babies (32.0% vs. 10.6%, *p* < 0.001), Table 2.

The babies' birth weight varied from 1.2 to 4.6 Kg, with a mean birth weight of 3.2 ± 3 Kg and 80.7% (1,422) of the babies were born with normal birth weight (2.5–3.9 Kg). Whereas 25.5% of low‐weight and 15.0% of overweight babies were admitted to the NICU, babies with low birth weight were more likely to be admitted to the NICU (*p* < 0.001). Male babies constituted 52.6% (1422/1762) of the babies born and concerning NICU admission, 12.5% of them were admitted to the unit and this was significant (*p* = 0.003) when compared to their female counterparts. Birth asphyxia at birth was assessed to be severe in 3.2% (56) and moderate in 32.2% (567) of the babies, of which 50.0%^13^ and 78.3% (444) of these babies were admitted to the NICU respectively. Five minutes after delivery, the rate of asphyxia reduced to 2.0%^14^ for severe asphyxia and to 7.0% (124) for moderate asphyxia. Similarly, 30.6%^11^ and 66.1% (82) of these asphyxic babies were respectively admitted to the NICU for management. Severe asphyxia, whether diagnosed at birth or 5 min after delivery was significantly associated with admission to the NICU and these babies respectively had 15.8 and 12.1 odds of being admitted when compared to the non‐asphyxiated babies. On the other hand, moderate asphyxia at birth but not those diagnosed 5 min after delivery was significantly associated with NICU admission and this group of babies had 4.8 risks of being admitted when compared to their non‐asphyxiated counterparts. Birth defects, even though significantly associated with NICU admission were only detected among 12 of the babies (*p* = 0.028), Table 3. Although 8.0% (142/1777) of the deliveries were diagnosed to be complicated, 11.2% (197/1766) of mothers were diagnosed with medical conditions before delivery, but none of these factors had significant association with the babies' admission to the NICU, Table 4.

**Table 3:** Babies' birth characteristics and admission to the neonatal intensive care unit

Variables	Total N (%)	Not admitted to NICU N (%) *ref*	Admitted to NICU N (%)	Exp (CI) (95%)	*p* Value
Baby weight in Kg					
<2.5	220 (12.5)	164 (74.5)	56 (25.5)	1.080 (0.528–2.212)	<0.001
2.5–3.9	1422 (80.7)	1315 (92.5)	107 (7.5)	0.309 (0.162–0.587)	0.833
>4.0 ***(ref)***	120 (6.8)	102 (85.0)	18 (15.0)		
Total	1762 (100)	1581 (89.7)	181 (10.3)		
Baby sex					
Male	928 (52.6)	810 (87.3)	118 (12.7)	1.828 (1.235–2.705)	0.003
Female ***(ref)***	836 (47.4)		65 (7.8)		
Total	1764 (100)	1580 (89.6)	183 (10.4)		
Apgar at 1 min					
0–3	56 (3.2)	28 (50.0)	28 (50.0)	15.772 (3.926–63.362)	<0.001
4–7	567 (32.2)	444 (78.3)	123 (21.7)	4.783 (3.042–7.519)	<0.001
>7 ***(ref)***	1140 (64.7)	1106 (97.0)	34 (3.0)		
Total	1763 (100)	1578 (89.5)	185 (10.5)		
Apgar at 5 min					
0–3	36 (2.0)	25 (69.4)	11 (30.6)	12.092 (7.250–20.168)	<0.001
4–7	124 (7.0)	42 (33.9)	82 (66.1)	0.540 (0.114–2.554)	0.437
>7 ***(ref)***	1605 (90.9)	1513 (94.3)	92 (5.7)		
Total	1765 (100)	1580 (89.5)	186 (10.5)		
Presence of birth abnormalities/injuries					
No	1762 (99.3)	1581 (89.7)	181 (10.3)	0.17 (0.035–0.830)	0.028
Yes ***(ref)***	12 (0.7)	8 (66.7)	4 (33.3)		
Total	1774 (100)	1589 (89.6)	185 (10.4)		

Abbreviation: (***ref***), reference category.

**Table 4:** Labor complications and mothers' medical history

Variable	Total	Not admitted to NICU N (*ref)*	Admitted to NICU N	Exp (CI) (95%)	*p* Value
Complications during delivery					
None	1635 (92.0)	1483	152	0.512 (0.246–1.069)	0.075
Obstetric hemorrhage	27 (1.5)	22	5	1.136 (0.340–3.797)	0.835
Preeclampsia	14 (0.8)	9	5	2.778 (0.752–10.260)	0.125
Fetal distress	35 (2.0)	26	9	1.731 (0.610–4.909)	0.302
Obstructed labor ***(ref)***	54 (3.0)	45	9		
Others	12 (0.7)	7	5	3.571 (0.924–13.811)	0.065
Total	1777 (100)	1592	185		
Medical condition					
None	1569 (88.8)	1403	166	0.673 (0.193–2.352)	0.535
Hepatitis B	158 (8.9)	148	10	0.330 (0.080–1.362)	0.125
Syphilis	19 (1.1)	18	1	0.287 (0.026–3.126)	0.306
HIV/AIDS ***(ref)***	20 (1.1)	17	3		
Total	1766 (100)	1586	180		

Abbreviation: (***ref**)*, reference category.

## 4 DISCUSSION

This study sought to establish the factors associated with NICU admission in St. Theresa's hospital located in the Nandom Municipality of the Upper West Region of Ghana. The overall NICU admission rate of 10.4% appears to be lower when compared to earlier studies where NICU admission rates were found to be between 11.1% to 19.8%.^3^, ^6^, ^10^, ^12^

As expected, the ages of the mothers reviewed in this study ranged from 13 to 48 years which is within women in fertility age (WIFA). However, slightly over 10% of the babies were borne by teenage mothers, yet there was no significant association between teenage pregnancy and admission to NICU in contrast with other findings where teenage pregnancy was found to be one of the major associated factors to NICU admission.^15^ Similarly, although some studies have observed an increased risk of NICU admission of infants born to mothers of advanced age,^16^, ^17^, ^18^ this study did not find significant association between advanced maternal age and admission to NICU, a finding similar to the observation made by Wang et al.^19^

ANC attendance was found to be associated with NICU admission in our study. Though our review showed that nine out of every 10 women achieved the recommended minimum of 4 ANC attendance per pregnancy, mothers whose ANC attendance was less than four sessions were twice likely to have their babies admitted to the NICU. This is similar to an observation made by Manjavidze et al.^20^ in their study in Georgia who found that women who had inadequate ANC attendance had an increased chance of having their babies admitted to NICU. Possibly, the absence from ANC education could have obscured the early detection and prevention of adverse pregnancy outcomes among such mothers.^21^

Parity, Mode of delivery, gestational age and multiple gestations were observed to be associated with NICU admission. Infants of nulliparous mothers were about three times more likely to be admitted to NICU as compared to other mothers. Clapp et al.^22^ reported similar findings where nulliparity was observed to have higher odds for admission to NICU. This observation is further supported by Kakoma et al. who concluded that nulliparity was associated with a high risk of admission to NICU in Rwandan district hospitals.^23^ However, our study did not seek to explain how nulliparity increases the risk of NICU admission. Conversely, there was no significant association between the gravidity of mothers and admission to NICU, contradicting other studies that found gravidity as a risk factor for NICU admission.^24^, ^25^

Multiple pregnancy was identified as a significant contributor to the admission of neonates to the NICU. Some studies support the assertion that multiple pregnancy is a risk factor for NICU admission.^26^ A study done in Beijing by Su et al.^27^ also supports this finding that multiple pregnancy increases the risk of NICU admission. Similarly, the increased risk of NICU admission among mothers with multiple pregnancy is supported by a study on multiple gestations conducted by Refuerzo et al.^28^ in the United States of America.

Prematurity was found to have a strong association with admission to NICU from our study. Babies who were born before 37 weeks of gestation were about four times more likely to be admitted to NICU as compared to those who were born at term. Desalew et al.^7^ found preterm delivery as an independent risk factor for admission to NICU. This is demonstrated in the results of this study and similar findings shown by Tette et al.^11^ From the results of this study, babies also born after 42 weeks of gestation showed an increased risk of admission to NICU. These babies were about five times more likely to be admitted to NICU as compared to those born at term, as reported by Linder et al.^29^

As per the findings of our study, there was an increased risk of NICU admission following cesarean section and instrumental delivery. Babies born by cesarean section were two times more likely to be admitted to NICU as compared to those who delivered by spontaneous vaginal delivery (SVD). This finding is comparable to the results from a study conducted by Fallah et al.^12^ who showed that infants delivered by cesarean section were more likely to be admitted to NICU. More significantly, deliveries conducted instrumentally were five times more likely to result in NICU admission when compared to SVD. This study showed that both cesarean section and instrumental delivery influence NICU admission. A study by Sowemimo et al.^13^ showed that out of 66 deliveries conducted instrumentally, 45.5% resulted in admission to NICU, also, the risk of adverse neonatal outcome was increased in mothers who had cesarean section.^30^ Undoubtedly, there could exist some confounding factors beyond the scope of the current study.

Neonatal factors such as birth weight, sex, asphyxia, and congenital defects were found to be associated with admission to the NICU. Barker et al.^31^ in their study showed the link between low birth weight and an increased risk of comorbidities later in life. The incidence of low birth weight could be due to inadequate nutrition by the mother during pregnancy, iron or folate deficiency and low socioeconomic status.^32^ Low birth weight, among other intrapartum and neonatal factors is an important predictor of neonatal mortality.^7^ A study conducted in Jordan by Khasawneh et al.^6^ observed that 39% of babies admitted to NICU had low birth weight with similar findings reported by Ali et al in Pakistan.^33^ Demisse et al.^34^ in their study in Ethiopia also observed an increased rate of NICU admission among low birth weight infants with a comparable admission rate of 35% making up NICU admissions.

The results of our study show a slight preponderance towards the male child being admitted to NICU. The males were almost twice likely to be sent to NICU as compared to their female counterparts. Similar findings were reported by Desalew et al.^7^ in their study. Rachakonda et al.^10^ also reported an increased number of males admitted to NICU compared to females. Similarly, a national cohort survey in Taiwan conducted by Weng et al.^35^ concluded that generally, the male gender carried higher risks of adverse neonatal outcomes like preterm delivery, congenital anomaly and operative delivery among others. Some studies have proposed explanations as to the difference in morbidity in male and female neonates and have suggested that the male sex appeared to be more at risk due to factors including low Apgar score, intrauterine growth retardation, respiratory insufficiency, or prematurity.^36^ However, the underlying mechanisms contributing to this observation have not been pointed out.^14^

According to the results of our study, severe and moderate birth asphyxia were found to be associated with an increased risk for admission to the NICU. After a minute following delivery, babies who recorded Apgar score below 3 were 15 times more likely to be admitted to NICU. Those who were moderately asphyxiated at 1 min were four times as likely to be admitted to NICU as compared to those with mild asphyxia. Birth asphyxia has been shown to increase the likelihood of neonatal morbidity and mortality.^37^ A study conducted by Gauchan et al.^38^ also found perinatal asphyxia to be a cause for admission to NICU.

## 5 CONCLUSION

In conclusion, the prevalence of NICU admission was 10.4%. Mothers who attended less than four antenatal sessions had an increased risk of having their babies admitted to NICU. The study found that nulliparity and multiple pregnancy were risk factors for the admission of babies to NICU. Also, preterm babies and those born post‐term had an increased risk of admission to NICU, likewise babies delivered by cesarean section and instrumental delivery. Low birth weight babies, male babies, Apgar score, and birth asphyxia were associated with admission of babies to the NICU. These factors could provide the bases for healthcare providers to put in place preventive strategies aimed at reducing neonatal admissions in the study area.

## AUTHOR CONTRIBUTIONS

**Maroun Soribang Ziem**: Data curation. **Fidelis Adam Saaka**: Data curation. **Ezekiel Kofi Vicar**: Data curation. **Eugene Dogkotenge Kuugbee**: Resources. **Akosua Bonsu Karikari**: Writing – review & editing. **Sebastian Yidana Ninimiya**: Resources. **Juventus Benogle Ziem**: Writing – review & editing. **Williams Walana**: Formal analysis; Writing – original draft.

## CONFLICT OF INTEREST

The authors declare no conflict of interest.

## FUNDING

This study did not receive any external funding.

## TRANSPARENCY STATEMENT

The lead author Williams Walana affirms that this manuscript is an honest, accurate, and transparent account of the study being reported; that no important aspects of the study have been omitted; and that any discrepancies from the study as planned (and, if relevant, registered) have been explained.

## Data Availability

The data set for this study is available with the corresponding author, and will be made available upon reasonable request. The corresponding author has full access to all of the data in this study and takes complete responsibility for the integrity of the data and the accuracy of the data analysis.
